# Supplementary material for: Multifunctional Roles of Medicinal Plants in the Meat Industry: Antioxidant, Antimicrobial, and Color Preservation Perspectives
Source: Plants (Basel). 2025 Sep 2;14(17):2737. doi: 10.3390/plants14172737 (PMC12430295; doi:10.3390/plants14172737)
Supplement: Supplementary file 1 [file plants-14-02737-s001.zip › Supplementary materials Figure S1.pdf]

Supplementary materials Figure S1

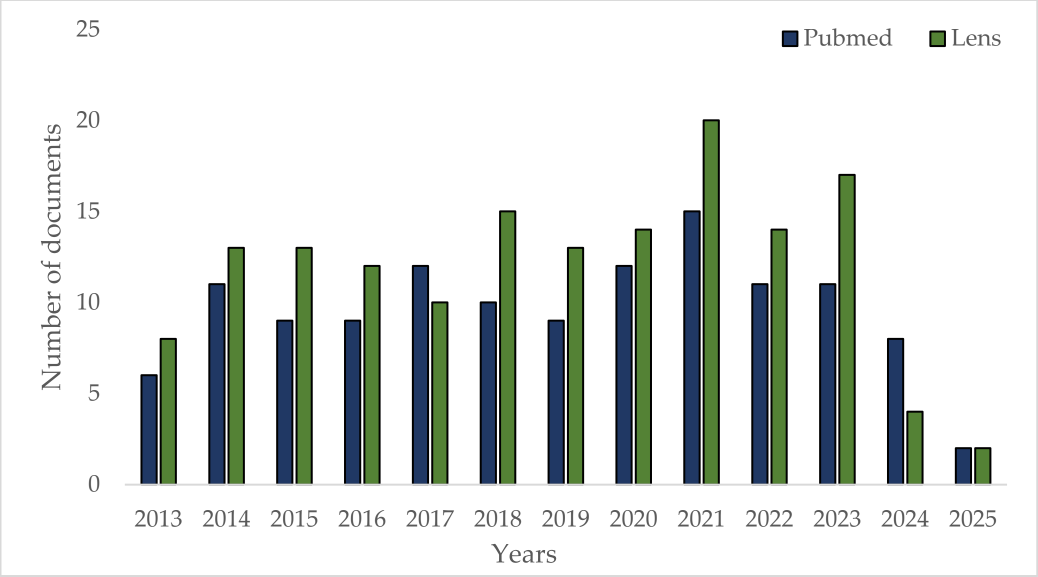

Figure S1. Publications trend of medicinal plants used in meat industry (based on data retrieved from Pubmed and Lens databases).
