# Supplementary material for: Multifunctional Roles of Medicinal Plants in the Meat Industry: Antioxidant, Antimicrobial, and Color Preservation Perspectives
Source: Plants (Basel). 2025 Sep 2;14(17):2737. doi: 10.3390/plants14172737 (PMC12430295; doi:10.3390/plants14172737)
Supplement: Supplementary file 1 [file plants-14-02737-s001.zip › Supplementary materials Table S1.pdf]

Table S1. The main chemical structures identified in medicinal plants

**Flavonoids**			
Apigenin	Quercetin	Kampherol	
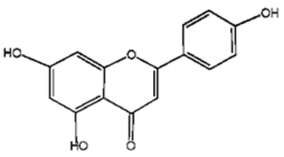	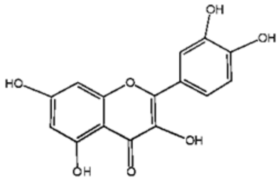	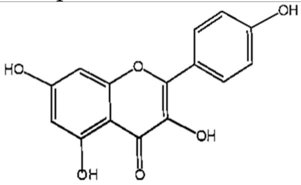	
Catechin	Cyanidin-3-Glucoside	Delphinidin-3-Glucoside	
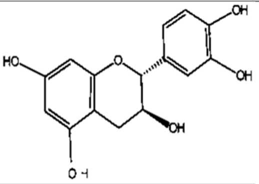	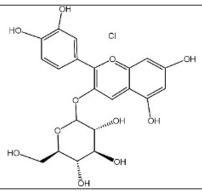	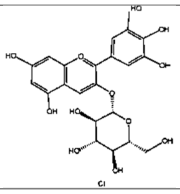	
P**olyphenol** (**diarylheptanoid**) - Curcumin			
	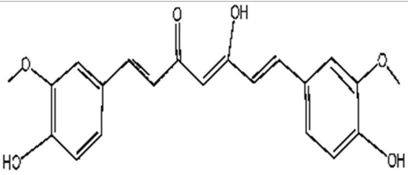		
**Phenolic acids**			
Caffeic Acid	Chlorogenic Acid	Gallic acid	
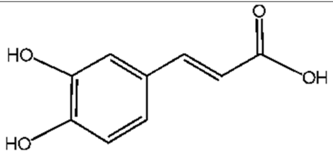	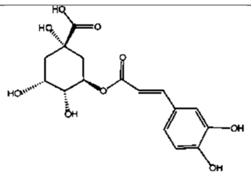	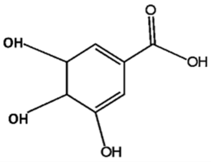	
**Alkaloids**			
Caffeine	Nigellidine	Piperine	
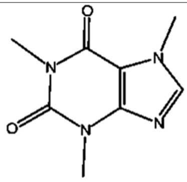	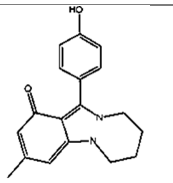	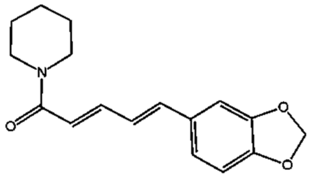	
**Essential oils**			
Gingerol	α-turmerone	Nigellone	Thymol
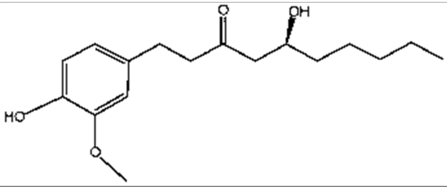	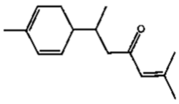	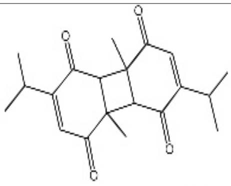	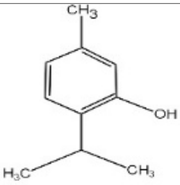

Chemical compound structure was drawn via the KingDraw-Free Chemical Structure Editor tool, (https://revvitysignals.com/products/research/chemdraw) accessed on 01 November 2024.
